# Predictors of Epstein-Barr virus serostatus in young people in England

**DOI:** 10.1186/s12879-019-4578-y

**Published:** 2019-11-28

**Authors:** Joanne R. Winter, Graham S. Taylor, Olivia G. Thomas, Charlotte Jackson, Joanna E. A. Lewis, Helen R. Stagg

**Affiliations:** 1 Centre for Molecular Epidemiology and Translational Research, Institute for Global Health, University College London, London, UK; 2 Institute of Immunology and Immunotherapy, College of Medical and Dental Sciences, University of Birmingham, Birmingham, UK; 3 Current address: MRC Clinical Trials Unit, University College London, London, UK; 4 National Institute for Health Research (NIHR) Health Protection Research Unit in Modelling Methodology, and Medical Research Council Centre for Outbreak Analysis and Public Health, Department of Infectious Disease Epidemiology, Imperial College London, London, UK; 5 Usher Institute of Population Health Sciences and Informatics, University of Edinburgh, Edinburgh, UK

**Keywords:** Epstein-Barr virus, Serostatus, Infectious mononucleosis, Cancer, Transmission, Risk factors

## Abstract

**Background:**

Epstein-Barr virus (EBV) is an important human pathogen which causes lifelong infection of > 90% people globally and is linked to infectious mononucleosis (arising from infection in the later teenage years) and several types of cancer. Vaccines against EBV are in development. In order to determine the most cost-effective public health strategy for vaccine deployment, setting-specific data on the age at EBV acquisition and risk factors for early infection are required. Such data are also important to inform mathematical models of EBV transmission that can determine the required target product profile of vaccine characteristics. We thus aimed to examine risk factors for EBV infection in young people in England, in order to improve our understanding of EBV epidemiology and guide future vaccination strategies.

**Methods:**

The Health Survey for England (HSE) is an annual, cross-sectional representative survey of households in England during which data are collected via questionnaires and blood samples. We randomly selected individuals who participated in the HSE 2002, aiming for 25 participants of each sex in each single year age group from 11 to 24 years. Stored samples were tested for EBV and cytomegalovirus (CMV) antibodies. We undertook descriptive and regression analyses of EBV seroprevalence and risk factors for infection.

**Results:**

Demographic data and serostatus were available for 732 individuals. EBV seroprevalence was strongly associated with age, increasing from 60.4% in 11–14 year olds throughout adolescence (68.6% in 15–18 year olds) and stabilising by early adulthood (93.0% in those aged 22–24 years). In univariable and multivariable logistic regression models, ethnicity was associated with serostatus (adjusted odds ratio for seropositivity among individuals of other ethnicity versus white individuals 2.33 [95% confidence interval 1.13–4.78]). Smoking was less strongly associated with EBV seropositivity.

**Conclusions:**

By the age of 11 years, EBV infection is present in over half the population, although age is not the only factor associated with serostatus. Knowledge of the distribution of infection in the UK population is critical for determining future vaccination policies, e.g. comparing general versus selectively targeted vaccination strategies.

## Background

Epstein-Barr Virus (EBV) is a herpesvirus that infects 90–95% of humans, causing lifelong infection [1, 2]. EBV infection during childhood is generally asymptomatic, however acquisition of EBV during adolescence or early adulthood often causes infectious mononucleosis (IM), [3] which can cause substantial morbidity during important educational periods in adolescents and young adults [4, 5]. EBV is associated with 1% of global cancers, particularly Hodgkin’s lymphoma, Burkitt’s lymphoma, nasopharyngeal cancer and gastric cancer [6].

EBV infection is not currently treatable nor preventable by vaccination; however, vaccine candidates are in development. In phase II trials, a first-generation vaccine administered to healthy seronegative volunteers aged 16–25 years demonstrated protection against IM but not EBV infection [7]. Second-generation vaccines elicited higher levels of antibody responses in animal models, [8] and first-in-human trials are likely to begin soon. Mathematical modelling of different vaccination strategies is essential to determine the effectiveness and cost-effectiveness of different vaccination strategies for reducing rates of EBV infection, IM, and EBV-associated cancers, taking into account factors such as vaccine efficacy, duration of protection and differing outcomes according to age at infection.

A greater understanding of EBV epidemiology, including the dynamics of EBV infection in different sub-populations, is necessary for the development of such models. EBV seroprevalence increases with age; 90–95% of people globally are infected by age 25, whilst 5–10% remain seronegative throughout life [9]. The best public health strategy for the deployment of an infection-preventing vaccine may vary between settings; infection appears to occur at younger ages in resource-limited countries and thus children will need to be vaccinated early [10–12]. However, if the duration of vaccine-induced protection is not lengthy, vaccinated individuals may become susceptible to natural infection at an age where the consequences of infection are more severe, for example leading to IM or cancer [13].

Additionally, sub-optimal vaccine coverage even of a vaccine with a long duration of protection will lead to a higher age at infection amongst those who remain unvaccinated. In such situations it may be better to delay vaccination until the pre-teenage years, targeting individuals who remain EBV seronegative. Alternatively, a vaccine protecting against IM and EBV-associated diseases (such as certain cancers) could be administered to older children as they approach adolescence, which may be effective even with a shorter duration of protection. After the licensing of vaccine candidates, strategic discussions will need to take place nationally and be informed by accurate national data on the epidemiology of EBV infection.

In the United Kingdom, EBV seroprevalence increases rapidly in very young children, reaching 21 and 51% by the age of two years in children of white and Pakistani ethnicity, respectively [14]. Another study showed that EBV seroprevalence then remained relatively constant, at around 55%, between the ages of five and 11 years [15]. EBV seroprevalence was estimated at 75% in university students at 19 years and 92% by the age of 22 years [16]. We recently published summary data on the seroprevalence of EBV in adolescents in England [13]; however, to date no study has investigated factors associated with seropositivity that could inform a targeted vaccination strategy.

Our aim was to investigate the sociodemographic and lifestyle factors, particularly age, associated with EBV serostatus in children and young adults in England, and to discuss the implications of our findings for future EBV vaccination policy.

## Methods

### Study population

The Health Survey for England (HSE) is an annual, cross-sectional, representative survey of households in England. Its methods are described in detail elsewhere [17]. For this study, and in order to parameterise a model of EBV transmission, [13] we randomly selected individuals who participated in the 2002 HSE; 2002 was the most recent year in which survey participants gave consent for future studies to test their blood samples for blood-borne viruses. Our aim was to include 25 participants of each sex in each single year age group from 11 to 24 years, in order to fill a gap in the literature and capture the years at which infection is most likely to have clinical consequences. The participant IDs were selected randomly by the HSE, however it was not possible at the time of sampling to determine whether the samples had already been used. As a result, more than 25 IDs were selected for each age-sex group to ensure there were sufficient samples for our analysis, and therefore there are not exactly 25 samples in each group (Additional file 1: Table S1).

### Measuring seroprevalence of Epstein-Barr virus and cytomegalovirus infection

Stored blood serum samples collected between January 2002 and March 2003 were obtained from the HSE. Samples were posted to the laboratory within two days, where they were centrifuged, and the remaining serum was frozen and stored at − 40°c until they were analysed, which was completed in September 2017 [18].

EBV virus capsid antigen (VCA)-specific IgG and CMV-specific IgG were detected in serum samples using commercial ELISA kits obtained from EUROIMMUN, Germany (EI2791–9601-G, EI2570-9601G). Assays were performed according to manufacturer’s instructions and serum antibody concentrations were calculated using a standard curve. Data on the performance of the assays are detailed in Additional file 1: Table S2. Results were presented in relative units (RU/mL); <16RU/mL samples were considered negative, ≤16 to <22RU/mL borderline and ≥ 22RU/mL positive. Borderline results from the EBV VCA IgG ELISA were subsequently subjected to re-analysis with an EBV immunoblot assay (EUROIMMUN, Germany, DY2790G) which revealed all borderline serum samples (*n* = 5) had reactivity to alternative EBV antigens; they were therefore considered seropositive.

### Statistical analysis

Data were analysed in Stata version 15.0. We weighted our sample, using the *svy* commands in Stata, to be representative of the English population in 2002 with respect to age and sex, utilising data from the Office for National Statistics [19]. All stated percentages are weighted. Descriptive analyses of the study population were undertaken. ArcMap 10.3.1 was used to create a map of EBV seroprevalence by English Government Office Region [20].

To investigate factors associated with being seropositive for EBV, we undertook logistic regression modelling. A causal inference framework was used to determine *a priori* factors to be included in multivariable models, from the available data collected in the HSE. We built two multivariable regression models.

A ‘whole-population’ model, which included our entire study population, examined the following factors: age, sex, ethnicity (categorised as ‘white’ or ‘other’ due to small numbers of non-white participants), body mass index (BMI; categorised as ‘underweight’ [BMI < 20], ‘healthy weight’ [20-<25], ‘overweight’ [25-<30]or ‘obese’ [≥30]), region of England and CMV serostatus.

A second ‘adults-only’ model was restricted to individuals aged ≥16 years, and additionally included factors for which data was only available for adults; smoking status (never smoked, current smoker, smoked in past) and occupational category from the National Statistics Socio-economic classification (NS-SEC) [21]. The NS-SEC categorises occupations into higher managerial and professional roles (involving strategy/supervision), intermediate occupations (typically clerical, sales, service or technical positions which do not involve general planning or supervision), routine and manual occupations (involving basic labour), never worked or long-term unemployed, and other. We excluded individuals missing data on one or more variables.

Planned sensitivity analyses investigated the impact of excluding CMV serostatus as a predictor of EBV serostatus, and the impact of classifying the originally indeterminate serological results as seronegative rather than seropositive.

### Ethical approval

This study was approved by the University College London Research Ethics Committee (5683/002). The HSE obtained informed written consent for blood samples to be collected and stored for future analyses [17].

## Results

Our study sample included 732 individuals aged 11–24 years, of whom 547 (74.6%) were EBV-seropositive. The characteristics of seropositive individuals are shown in Table 1.

**Table 1:** The number and weighted percentage of individuals seropositive for EBV in England in 2002

	Whole cohort		Adults only	
		EBV seropositive		EBV seropositive
Variable	Total number	Number (weighted %)	Total number	Number (weighted %)
Total	732	547 (74.6)	472	389 (74.6)
Sex				
Men	364	266 (72.5)	234	185 (78.8)
Women	368	281 (76.6)	238	204 (86.0)
CMV serostatus				
CMV-seronegative	557	406 (72.6)	346	275 (79.6)
CMV-seropositive	175	141 (80.9)	126	114 (90.5)
Age last birthday				
11–14 years	208	125 (60.4)		
15–18 years†	212	145 (68.6)	160	112 (70.1)
19–21 years	156	132 (84.6)	156	132 (84.6)
22–24 years	156	145 (93.0)	156	145 (93.0)
Ethnic group				
White	655	482 (73.4)	425	345 (81.2)
Other	77	65 (84.2)	47	44 (93.6)
BMI				
Healthy weight	418	300 (71.6)	259	207 (79.8)
Underweight	60	47 (78.7)	60	47 (78.7)
Overweight	141	113 (79.6)	102	90 (88.3)
Obese	87	65 (74.8)	35	31 (89.1)
Missing	26	22 (84.6)	16	14 (87.5)
Region				
East of England	78	50 (64.6)	52	39 (75.6)
North East	34	27 (79.8)	25	23 (91.8)
North West	130	98 (74.4)	80	66 (82.0)
Yorkshire and The Humber	82	70 (85.0)	58	51 (87.9)
East Midlands	74	57 (77.8)	48	39 (81.0)
West Midlands	70	46 (65.3)	33	24 (73.0)
London	63	50 (78.6)	42	37 (88.2)
South East	119	88 (73.4)	82	64 (78.2)
South West	82	61 (75.9)	52	46 (88.9)
Ever smoked^a^				
Never smoked	–	–	186	137 (73.8)
Currently smoke	–	–	134	124 (92.5)
Smoked in past	–	–	147	124 (84.4)
Missing	–	–	5	4 (80.0)
Occupational category^a^				
Higher managerial and professional	–	–	83	71 (85.9)
Intermediate occupations	–	–	69	61 (88.3)
Routine and manual occupations	–	–	254	202 (79.6)
Never worked or long-term unemployed	–	–	11	10 (91.2)
Other	–	–	55	45 (81.4)

^a^Adults aged ≥16 years only (*n* = 472). †16–18 years for ‘adult-only’ model. Percentages account for the weighting of the sample to be representative of the English population in 2002 with respect to age and sex. BMI: body mass index, *CI* confidence interval, *CMV* cytomegalovirus, *EBV* Epstein-Barr virus

**Fig. 1:**
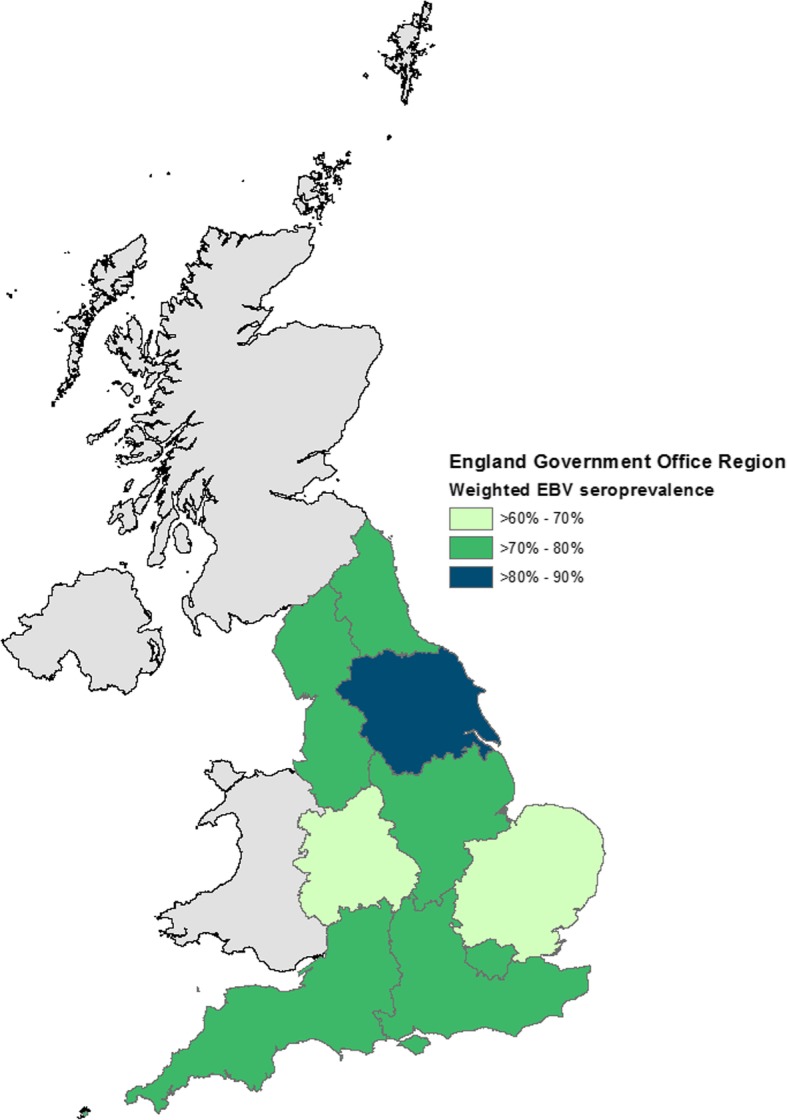
Weighted Epstein-Barr virus seroprevalence by English Government Office Region in 2002. Contains National Statistics data© Crown copyright and database right [2011] Contains public sector information licensed under the Open Government Licence v3.0

**Fig. 2:**
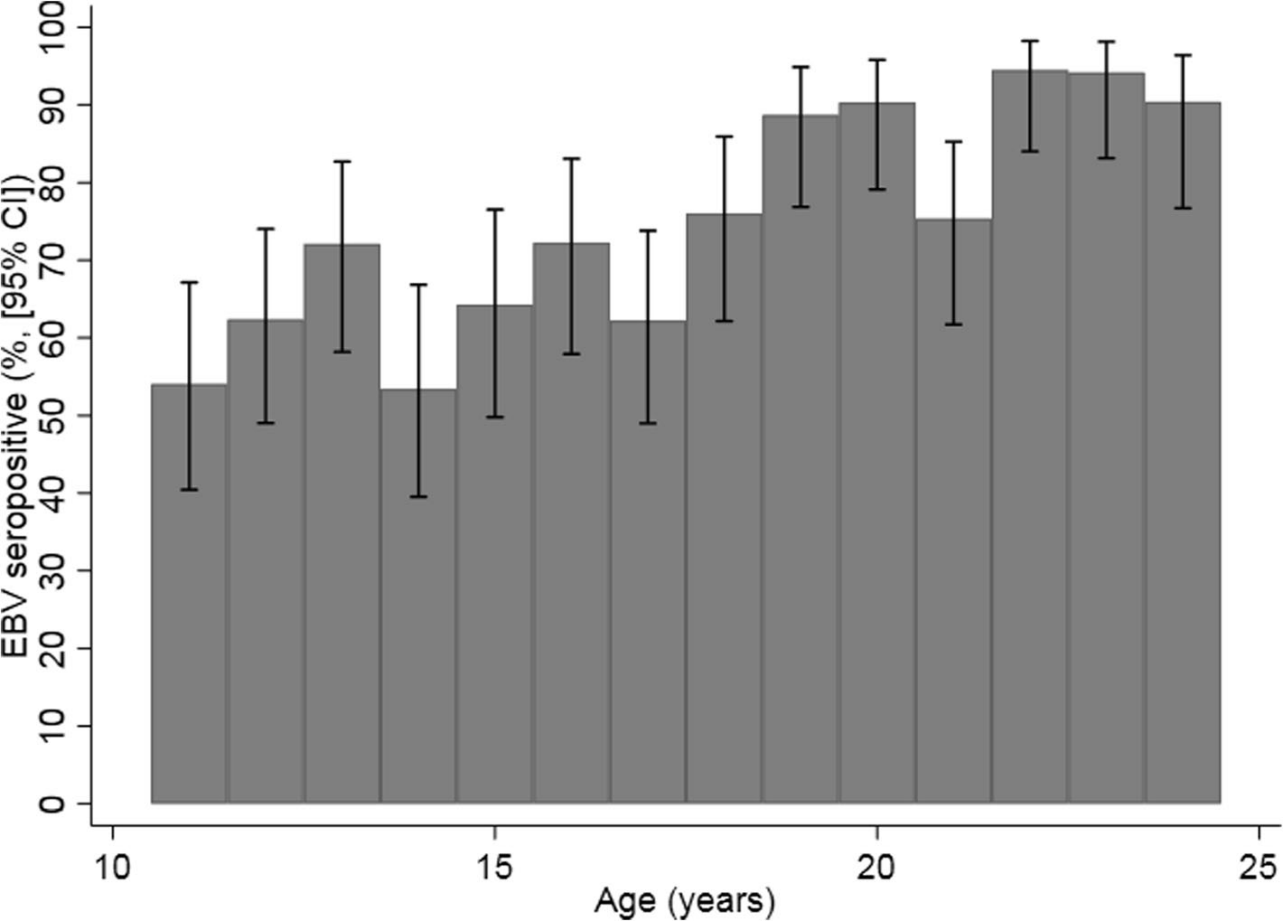
Weighted seroprevalence of Epstein-Barr virus by age in England in 2002. CI: confidence interval, EBV- Epstein Barr Virus

EBV serostatus was associated with CMV serostatus; 72.6% of CMV-seronegative individuals were EBV seropositive compared to 80.9% CMV-seropositive individuals (χ^2^ test *P* = 0.04, Table 1). Considerable variation in EBV seroprevalence was observed by UK region (Fig. 1, Table 1). EBV seropositivity increased with age, from 39.6% at 11–14 years to 93.0% at 22–24 years (Fig. 2).

Factors associated with EBV seropositivity were largely consistent between the univariable and multivariable models (Table 2). Increasing age was associated with increased EBV seroprevalence (adjusted odds ratio [aOR] 9.16 [95% confidence interval (CI) 4.38–19.14] for people aged 22–24 years compared to those aged 11–14 years), as was non-white ethnicity (aOR 2.33 [1.13–4.78]). CMV seropositivity was associated with EBV seropositivity in the ‘adults-only’ multivariable model (aOR 2.16 [1.05–4.43]) but not in the ‘whole population’ model (aOR 1.25 [0.79–1.98]).

**Table 2:** Univariable and multivariable logistic regression models of factors associated with Epstein-Barr Virus seropositivity in England in 2002

	Univariable		Multivariable (whole population)		Multivariable (adults only)	
	OR (95% CI)	*P* value	aOR (95% CI)	*P* value	aOR (95% CI)	*P* value
Sex						
Male	1.00		1.00		1.00	
Female	1.24 (0.87–1.75)	0.230	1.15 (0.79–1.67)	0.454	1.47 (0.86–2.53)	0.156
Age group (years)						
11–14	1.00		1.00			
15–18†	1.43 (0.97–2.11)	0.068	1.52 (1.01–2.30)	0.046	1.00	
19–21	3.61 (2.13–6.13)	< 0.001	3.76 (2.16–6.57)	< 0.001	2.51 (1.31–4.82)	0.006
22–24	8.76 (4.30–17.83)	< 0.001	9.16 (4.38–19.14)	< 0.001	6.14 (2.64–14.27)	< 0.001
Ethnicity						
White	1.00		1.00		1.00	
Other	1.93 (1.04–3.60)	0.038	2.33 (1.13–4.78)	0.021	4.26 (1.03–17.58)	0.045
BMI						
Healthy weight	1.00		1.00		1.00	
Underweight	1.46 (0.77–2.77)	0.243	1.13 (0.59–2.17)	0.718	1.13 (0.55–2.32)	0.731
Overweight	1.55 (0.97–2.49)	0.070	1.16 (0.69–1.95)	0.567	1.93 (0.88–4.24)	0.101
Obese	1.18 (0.65–2.13)	0.588	1.25 (0.67–2.33)	0.483	1.47 (0.45–4.77)	0.517
Region of UK						
East of England	1.00		1.00		1.00	
North East	2.16 (0.73–6.43)	0.166	2.46 (0.83–7.30)	0.105	4.78 (0.98–23.19)	0.052
North West	1.59 (0.82–3.10)	0.169	1.92 (0.94–3.90)	0.072	1.28 (0.55–2.99)	0.570
Yorkshire and The Humber	3.11 (1.41–6.84)	0.005	3.11 (1.39–6.98)	0.006	1.90 (0.73–4.95)	0.187
East Midlands	1.92 (0.87–4.20)	0.104	2.38 (1.06–5.33)	0.035	1.80 (0.67–4.87)	0.243
West Midlands	1.03 (0.50–2.10)	0.934	1.34 (0.63–2.86)	0.450	0.96 (0.28–3.26)	0.946
London	2.01 (0.90–4.52)	0.090	1.42 (0.61–3.29)	0.415	1.10 (0.37–3.24)	0.866
South East	1.51 (0.76–3.03)	0.242	1.84 (0.89–3.79)	0.099	1.27 (0.53–3.03)	0.589
South West	1.73 (0.87–3.43)	0.118	1.97 (0.93–4.17)	0.077	2.84 (0.87–9.24)	0.082
CMV serostatus						
Seronegative	1.00		1.00		1.00	
Seropositive	1.59 (1.06–2.39)	0.026	1.25 (0.79–1.98)	0.337	2.16 (1.05–4.43)	0.036
Smoking status^a^						
Never smoked	1.00		–	–	1.00	
Current smoker	4.40 (2.22–8.73)	< 0.001	–	–	4.29 (2.13–8.65)	< 0.001
Smoked in past	1.92 (1.09–3.40)	0.025	–	–	1.94 (0.99–3.81)	0.054
Occupational category^a^						
Higher managerial and professional	1.00	< 0.001	–	–	1.00	
Intermediate occupations	1.23 (0.43–3.53)	0.695	–	–	1.54 (0.47–5.03)	0.469
Routine and manual occupations	0.64 (0.30–1.36)	0.245	–	–	1.41 (0.62–3.22)	0.416
Never worked or long-term unemployed	1.70 (0.19–14.82)	0.631	–	–	3.16 (0.22–45.85)	0.397
Other	0.72 (0.26–2.03)	0.533	–	–	1.93 (0.56–6.69)	0.297

^a^Adults aged ≥16 years only (*n* = 472). †16–18 years for ‘adult-only’ model. Odds ratios account for the weighting of the sample to be representative of the English population in 2002 with respect to age and sex. The ‘whole population’ multivariable model included age, sex, CMV serostatus, ethnicity, BMI and region of England. The ‘adults only’ multivariable model included all variables shown in the table. *aOR* adjusted odds ratio, *BMI* body mass index, *CI* confidence interval, *CMV* cytomegalovirus, *OR* unadjusted odds ratio

Among adults, EBV seropositivity was higher among those who currently smoked (aOR 4.29 [2.13–8.65]), than those who had never smoked. There was no evidence of associations between sex, BMI or occupational category and EBV serostatus.

In sensitivity analyses, we firstly excluded CMV serostatus as a predictor of EBV serostatus, and secondly we classed indeterminate serology results (*n* = 5) as seronegative rather than seropositive. Both sensitivity analyses showed results consistent with our main analyses (Additional file 1: Table S3, Table S4).

## Discussion

The importance of EBV as a cancer-causing pathogen has generated international interest in developing an anti-infection vaccine [22]. The cost-effectiveness of different strategies to deploy such vaccines will vary from setting to setting and is dependent on the epidemiology of the infection. For example, EBV’s association with IM means that vaccines that do not produce lifelong immunity may be better targeted towards subgroups which are likely to acquire infection in adolescence. In this observational study of factors associated with EBV seroprevalence among young people in England in 2002, we explored the distribution of seroprevalence by age and the sources of additional variability. We found a substantial increase in EBV seroprevalence with age among our sample population, associations with ethnicity and smoking, and a potential association with CMV seroprevalence.

A series of studies have demonstrated that EBV is generally acquired pre-adulthood, and that this varies between settings [12]. Our findings regarding smoking fit with the prevailing narrative that there is an association between EBV and socioeconomic status, rather than smoking being an independent risk factor [12]. Unfortunately, we did not have a good measure of socioeconomic status in our analysis; the NS-SEC does not account for familial socioeconomic status during childhood, which is probably more relevant to EBV seroprevalence than individual occupational status in young adults, and we were unable to measure socioeconomic status in children at all.

We found that EBV prevalence varied substantially between regions of the UK in univariable analyses and in the whole-cohort model, but not in the adults-only model, suggesting confounding between region and socioeconomic status. There was also a strong association between EBV seropositivity and ethnicities other than white, in both univariable and multivariable models. This may be the result of different mixing patterns (as people of ethnic minorities are more likely to live in larger households), different feeding practices, or residual confounding of socioeconomic status. CMV is another herpesvirus which infects a high proportion of the population from a young age, [23] and has also been associated with EBV in other settings [24, 25].

In England, EBV infects 55% of the population by the age of 12 [15]; i.e. prior to adolescence, when the risk of IM increases. Cost-effective deployment of a cheap, infection-preventing, vaccine with a lifelong duration of protection could thus likely involve targeting the early years. However, future vaccines may produce a shorter duration of immunity, potentially delaying infection and resulting in an increasing incidence of IM (and IM-associated cancers). This could be compounded by sub-optimal vaccine coverage increasing the average age at infection [26] and consequently potentially increasing rates of IM – similarly to how sub-optimal coverage of the MMR vaccine led to an increase in congenital rubella syndrome in Greece [27, 28].

In such a scenario, targeted vaccine deployment to the social groups who acquire infection later (when the likelihood of IM is higher) might be considered, possibly with repeated dosing if required. Such targeting could be informed by the risk factors detected within this analysis, and data such as those presented here should be considered in conjunction with the characteristics of the vaccine available when determining what a vaccine policy should look like. If a vaccine was cheap and effective, then universal coverage would be appropriate. If the duration of protection was short, it may be prudent to give repeat doses of the vaccine to people who pick up the infection at the youngest age, which is linked to ethnicity and likely to socioeconomic status. The use of an expensive vaccine could be stratified on the basis of who is most likely to suffer EBV-related disease after infection, which we have studied separately [29].

The limitations of our work include the age of the data and the use of a cross-sectional study design, preventing determination of the temporality of the correlation between EBV and CMV infection. In our analysis, EBV seroprevalence was higher than CMV seroprevalence in all age groups, and both increased with age. We found that CMV was associated with EBV in univariable analyses, and in the adults-only model, but not in the whole-cohort multivariable model. As both EBV and CMV are associated with increasing age, particularly during adolescence, we would not expect an association between CMV and EBV to persist in the whole-cohort multivariable model. It is possible that as the association between age and EBV seroprevalence was less strong in the adults-only multivariable model (as EBV seroprevalence starts to saturate as people reach adulthood), there was enough of a residual effect that the association between EBV and CMV could be detected. Unfortunately, our sample size was not large enough to investigate the interactions between EBV, CMV and age in more detail. The association may result from shared genetic, immunological and/or sociodemographic risk factors, or one infection could increase susceptibility to the other. Longitudinal studies with serial testing are necessary to explore this association, and additional risk factors, in more detail.

We elected to measure IgG antibodies to the EBV VCA protein and whole CMV virus, as these antibodies are present in all infected individuals and persist for life. Although we did not test for IgM antibodies, and cannot exclude the possibility that some seronegative individuals may have been recently infected, we note that VCA-specific IgG and IgM antibodies usually appear contemporaneously [30] and therefore we would expect the number of such individuals in our study to be low.

## Conclusions

Knowledge of the distribution of EBV infection among young population groups in England is critical for determining future vaccination policies, including the cost-effectiveness of general versus selective approaches. Data such as those presented here should be used together with detailed information on vaccine characteristics, the implications of remaining EBV-uninfected for life, the ramifications of delayed infection, and the financial costs of IM and EBV-associated cancers to inform such policies.

## Supplementary information

**Additional file 1: Table S1.** Distribution of study participants by age and gender. **Table S2.** ELISA assay performance.0020. **Table S3.** Sensitivity analysis of multivariable logistic regression models of factors associated with Epstein-Barr Virus seropositivity in England in 2002, excluding Cytomegalovirus serostatus as a risk factor. **Table S4.** Sensitivity analysis of multivariable logistic regression models of factors associated with Epstein-Barr Virus seropositivity in England in 2002, with indeterminate serology results reclassified as seronegative rather than seropositive.

## Data Availability

The data used in this study was under license from the Health Survey for England, and so are not publicly available, but can be requested from the HSE.

## Abbreviations

aOR: Adjusted odds ratio
BMI: Body mass index
CI: Confidence interval
CMV: Cytomegalovirus
EBV: Epstein-Barr virus
ELISA: Enzyme-linked immunosorbent assay
HSE: Health Survey for England
IM: Infectious mononucleosis
NS-SEC: National statistics socio-economic classification
RU: Relative units
VCA: Virus capsid antigen
