# Supplementary material for: Predictors of Epstein-Barr virus serostatus in young people in England
Source: BMC Infect Dis. 2019 Nov 28;19:1007. doi: 10.1186/s12879-019-4578-y (PMC6883578; doi:10.1186/s12879-019-4578-y)
Supplement: Supplementary file 1 — Additional file 1: Table S1. Distribution of study participants by age and gender. Table S2. ELISA assay performance.0020. Table S3. Sensitivity analysis of multivariable logistic regression models of factors associated with Epstein-Barr Virus seropositivity in England in 2002, excluding Cytomegalovirus serostatus as a risk factor. Table S4. Sensitivity analysis of multivariable logistic regression models of factors associated with Epstein-Barr Virus seropositivity in England in 2002, with indeterminate serology results reclassified as seronegative rather than seropositive. [file 12879_2019_4578_MOESM1_ESM.docx]

## Supplementary Materials

Additional file 1: Table S1: Distribution of study participants by age and gender

| Age (years) | Male | Female | Total |
| --- | --- | --- | --- |
| 11 | 25 | 26 | 51 |
| 12 | 26 | 26 | 52 |
| 13 | 26 | 27 | 53 |
| 14 | 27 | 25 | 52 |
| 15 | 26 | 26 | 52 |
| 16 | 27 | 27 | 54 |
| 17 | 26 | 30 | 56 |
| 18 | 25 | 25 | 50 |
| 19 | 26 | 27 | 53 |
| 20 | 26 | 25 | 51 |
| 21 | 26 | 26 | 52 |
| 22 | 26 | 29 | 55 |
| 23 | 26 | 26 | 52 |
| 24 | 26 | 23 | 49 |
| Total | 364 | 368 | 732 |

Additional file 1: Table S2: ELISA assay performance

|  | EBV VCA IgG | CMV IgG |
| --- | --- | --- |
| Code | EI 2791-9601 G | EI 2570-9601 G |
| Antigen | Purified EBV capsid antigen from B-cells infected with P3HR1 EBV strain. | MRC-5 cells infected with AD169 strain of CMV |
| Linearity | R^2^ > 0.95. Linear within the manufacturer’s specified concentration range (4-141 RU/ml) | R^2^ > 0.95. Linear within the manufacturer’s specified concentration range (10-149 RU/ml) |
| Reproducibility | Intra-assay variation 4.2-7.4% CV  Inter-assay variation 3.2-8.2% CV | Intra-assay variation 4.2-5.4% CV  Inter-assay variation 5.7-11.2% CV |
| Cross-reactivity | No cross-reactivity with 85 antibodies specific for 12 viruses | No cross-reactivity with 228 antibodies specific for 19 viruses |
| Interference | No influence on results by haemolytic, lipaemic or icteric samples. | No influence on results by haemolytic, lipaemic or icteric samples. |
| Specificity | 100% | 100% |
| Sensitivity | 100% | 99.2% |

The EBV and CMV ELISAs used in this work are commercially available CE marked validated assays. Manufacturer’s stated performance of the assay is described. Specificity and sensitivity were calculated using 175 (EBV) or 396 (CMV) clinically characterised patient samples – supplied by INSTAND, NEQAS, Labquality, MQ and RfB.

Additional file 1: Table S3 Sensitivity analysis of multivariable logistic regression models of factors associated with Epstein-Barr Virus seropositivity in England in 2002, excluding Cytomegalovirus serostatus as a risk factor.

|  | **Multivariable (whole population)** | **Multivariable (adults only)** |
| --- | --- | --- |
|  | **aOR (95% CI)** | **aOR (95% CI)** |
| **Sex** |  |  |
| **Male** | **1.00** | **1.00** |
| **Female** | **1.16 (0.80-1.68)** | **1.55 (0.91-2.63)** |
| **Age group (years)** |  |  |
| **11-14** | **1.00** |  |
| **15-18** | **1.55 (1.02-2.33)** | **1.00** |
| **19-21** | **3.86 (2.21-6.72)** | **2.43 (1.27-4.67)** |
| **22-24** | **9.31 (4.45-19.47)** | **5.69 (2.44-13.26)** |
| **Ethnicity** |  |  |
| **White** | **1.00** | **1.00** |
| **Other** | **2.51 (1.25-5.04)** | **5.56 (1.45-21.28)** |
| **BMI** |  |  |
| **Healthy weight** | **1.00** | **1.00** |
| **Underweight** | **1.13 (0.59-2.17)** | **1.11 (0.54-2.27)** |
| **Overweight** | **1.17 (0.70-1.96)** | **1.87 (0.85-4.13)** |
| **Obese** | **1.28 (0.68-2.39)** | **1.52 (0.47-4.90)** |
| **Region of UK** |  |  |
| **East of England** | **1.00** | **1.00** |
| **North East** | **2.38 (0.82-6.93)** | **4.48 (1.00-20.05)** |
| **North West** | **1.88 (0.93-3.77)** | **1.20 (0.51-2.83)** |
| **Yorkshire and The Humber** | **3.08 (1.38-6.88)** | **1.84 (0.70-4.86)** |
| **East Midlands** | **2.31 (1.04-5.13)** | **1.70 (0.63-4.60)** |
| **West Midlands** | **1.33 (0.63-2.83)** | **0.88 (0.27-2.88)** |
| **London** | **1.44 (0.62-3.34)** | **1.24 (0.40-3.80)** |
| **South East** | **1.79 (0.88-3.66)** | **1.21 (0.50-2.95)** |
| **South West** | **1.98 (0.93-4.20)** | **2.72 (0.84-8.77)** |
| **Smoking status*** |  |  |
| **Never smoked** |  | **1.00** |
| **Current smoker** |  | **4.38 (2.17-8.83)** |
| **Smoked in past** |  | **1.94 (0.98-3.83)** |
| **Occupational category*** |  |  |
| **Higher managerial and professional** |  | **1.00** |
| **Intermediate occupations** |  | **1.37 (0.42-4.46)** |
| **Routine and manual occupations** |  | **1.25 (0.54-2.89)** |
| **Never worked or long-term unemployed** |  | **2.71 (0.19-38.26)** |
| **Other** |  | **1.68 (0.50-5.70)** |

*Adults aged ≥16 years only (n=472). †16-18 years for ‘adult-only’ model. Odds ratios account for the weighting of the sample to be representative of the English population in 2002 with respect to age and sex. The ‘whole population’ multivariable model included age, sex, CMV serostatus, ethnicity, BMI and region of England. The ‘adults only’ multivariable model included all variables shown in the table. aOR: adjusted odds ratio, BMI: body mass index, CI: confidence interval.

Additional file 1: Table S4: Sensitivity analysis of multivariable logistic regression models of factors associated with Epstein-Barr Virus seropositivity in England in 2002, with indeterminate serology results reclassified as seronegative rather than seropositive.

|  | Multivariable (whole population) | Multivariable (adults only) |
| --- | --- | --- |
|  | aOR (95% CI) | aOR (95% CI) |
| Sex |  |  |
| Male | 1.00 | 1.00 |
| Female | 1.15 (0.79-1.67) | 1.47 (0.86-2.53) |
| Age group (years) |  |  |
| 11-14 | 1.00 |  |
| 15-18 | 1.52 (1.01-2.30) | 1.00 |
| 19-21 | 3.76 (2.16-6.57) | 2.51 (1.31-4.82) |
| 22-24 | 9.16 (4.38-19.14) | 6.14 (2.64-14.27) |
| Ethnicity |  |  |
| White | 1.00 | 1.00 |
| Other | 2.33 (1.13-4.78) | 4.26 (1.03-17.58) |
| BMI |  |  |
| Healthy weight | 1.00 | 1.00 |
| Underweight | 1.13 (0.59-2.17) | 1.13 (0.55-2.32) |
| Overweight | 1.16 (0.69-1.95) | 1.93 (0.88-4.24) |
| Obese | 1.25 (0.67-2.33) | 1.47 (0.45-4.77) |
| Region of UK |  |  |
| East of England | 1.00 | 1.00 |
| North East | 2.46 (0.83-7.30) | 4.78 (0.98-23.19) |
| North West | 1.92 (0.94-3.90) | 1.28 (0.55-2.99) |
| Yorkshire and The Humber | 3.11 (1.39-6.98) | 1.90 (0.73-4.95) |
| East Midlands | 2.38 (1.06-5.33) | 1.80 (0.67-4.87) |
| West Midlands | 1.34 (0.63-2.86) | 0.96 (0.28-3.26) |
| London | 1.42 (0.61-3.29) | 1.10 (0.37-3.24) |
| South East | 1.84 (0.89-3.79) | 1.27 (0.53-3.03) |
| South West | 1.97 (0.93-4.17) | 2.84 (0.87-9.24) |
| CMV serostatus | | |
| Seronegative | 1.00 | 1.00 |
| Seropositive | 1.25 (0.79-1.98) | 2.16 (1.05-4.43) |
| Smoking status* | | |
| Never smoked | - | 1.00 |
| Current smoker | - | 4.29 (2.13-8.65) |
| Smoked in past | - | 1.94 (0.99-3.81) |
| Occupational category* | | |
| Higher managerial and professional | - | 1.00 |
| Intermediate occupations | - | 1.54 (0.47-5.03) |
| Routine and manual occupations | - | 1.41 (0.62-3.22) |
| Never worked or long-term unemployed | - | 3.16 (0.22-45.85) |
| Other | - | 1.93 (0.56-6.69) |

*Adults aged ≥16 years only (n=472). †16-18 years for ‘adult-only’ model. Odds ratios account for the weighting of the sample to be representative of the English population in 2002 with respect to age and sex. The ‘whole population’ multivariable model included age, sex, CMV serostatus, ethnicity, BMI and region of England. The ‘adults only’ multivariable model included all variables shown in the table. aOR: adjusted odds ratio, BMI: body mass index, CI: confidence interval, CMV: cytomegalovirus.
